# Biological Activities of Three Essential Oils of the Lamiaceae Family

**DOI:** 10.3390/medicines4030063

**Published:** 2017-08-23

**Authors:** Gema Nieto

**Affiliations:** Department of Food Technology and Human Nutrition, Veterinary Faculty, University of Murcia, Espinardo, 30071 Murcia, Spain; gnieto@um.es

**Keywords:** aromatic plants, essential oil, food additives, antioxidant, antimicrobial

## Abstract

Herbs and spices have been used since ancient times to improve the sensory characteristics of food, to act as preservatives and for their nutritional and healthy properties. Herbs and spices are generally recognized as safe (GRAS) and are excellent substitutes for chemical additives. Essential oils are mixtures of volatile compounds obtained, mainly by steam distillation, from medicinal and aromatic plants. They are an alternative to synthetic additives for the food industry, and they have gained attention as potential sources for natural food preservatives due to the growing interest in the development of safe, effective, natural food preservation. Lamiaceae is one of the most important families in the production of essential oils with antioxidants and antimicrobial properties. Aromatic plants are rich in essential oils and are mainly found in the Mediterranean region, where the production of such oils is a profitable source of ecological and economic development. The use of essential oils with antimicrobial and antioxidant properties to increase the shelf life of food is a promising technology, and the essential oils of the Lamiaceae family, such as rosemary, thyme, and sage, have been extensively studied with respect to their use as food preservatives. Regarding the new applications of essential oils, this review gives an overview of the current knowledge and recent trends in the use of these oils from aromatic plants as antimicrobials and antioxidants in foods, as well as their biological activities, future potential, and challenges.

## 1. Introduction

New preferences for a minimum of processed food and recent changes in consumers’ concern about synthetic preservatives [1] have led to an increase in the use of natural preservatives in foods [2]. 

Plant extracts have been employed by humans for thousands of years in traditional medicine. The Lamiaceae family contains important aromatic plants used in traditional and modern medicine and in the food and pharmaceutical industries. Rosemary, thyme, oregano, and sage are the most popular plants in Spanish traditional remedies and are often used for the treatment of gastritis, infections, dermatitis, bronchitis, and inflammation. Particularly, rosemary and sage have been extensively studied for their antioxidative and antimicrobial activity. The vegetation of Southeast Spain (Murcia) is rich in aromatic plants, and the province of Murcia is a major producer and processor of medicinal herbs [3]. Rosemary and thyme are mostly exploited for the extraction of essential oils (EOs). Among the >3000 essential oils known, about 300 are of importance for the pharmaceutical, agronomic, and food industries.

EOs are volatile liquids, or semi-liquids, extracted from plants, usually by steam distillation [4]. They are limpid and rarely coloured, are soluble in organic solvents with a lower density than that of water, and are lipid soluble. They can be synthesized by all plant organs (flowers, leaves, stems, twigs, seeds, fruits, roots, wood, or bark) and are stored in secretory cells, cavities, canals, epidermic cells or glandular trichomes. EOs are complex mixtures of sesquiterpene and monoterpene hydrocarbons and alcohols, ketones, and aldehydes (their oxygenated derivatives). Essential oils may also encompass other chemical families like fatty acids, oxides, and sulphur derivatives [5].

EOs are of scientific and popular interest because (a) they may act synergistically with other techniques of preservation, (b) they are generally recognized as safe, and (c) they show antioxidant, antibacterial, antidiabetic, antimutagenic, non-toxigenic, and antimycotic properties which are promising for their use as bioactive compounds in different foods [6].

The main components of common essential oils can be classified into two structural families with respect to hydrocarbon skeleton: terpenoids, formed by the combination of two (monoterpene), three (sesquiterpene) or four (diterpene) isoprene units, and phenylpropanoids. Both terpenoid and phenylpropanoid families comprise phenolic compounds, sometimes identified as principal components of several EOs.

Plants synthesize essential oils for a variety of purposes, including protection of the plant against fungi and bacteria, allelopathic activity, defence against insects (terpenoids are known to repel animals and insects) and attraction of pollinators and dispersal agents to favour the dispersion of seeds and pollens (the aromas and scents of the plants are responsible for the attraction of insects by the essential oils, characterized by volatility and a strong odour). Essential oils can protect plants against viruses, bacteria, fungi, insects, and herbivores [7]. The major activities of essential oils are antimicrobial, sedative, anti-inflammatory, bactericidal, antiviral, antifungal (fungicidal), and preservative for foods.

There is also a growing interest in plant EOs as natural alternatives to synthetic additives. This review describes the biological activities of EOs from the Lamiaceae family, primarily the antioxidant and antimicrobial activity of essential oils and the structure–activity relationships and mechanisms of action of their active components. Finally, an overview will be provided of other preventive and therapeutic properties of EOs, such as anti-angiogenic and anti-tumour properties.

## 2. Biological Activities

### 2.1. Antimicrobial Activity

The antibacterial activity of essential oils has been widely reported by Hammer et al. [8] and Dorman and Deans [9]. These authors reported that phenolic compounds present in essential oils, for example, carvacrol, eugenol, and thymol (from various plant origins), are active against many microorganisms. They show different activities against Gram-negative and Gram-positive bacteria, mainly due to the position of the hydroxyl group in the phenolic structure of the terpenes [8]. The main mechanism of antibacterial activity is the denaturation of proteins. An increase in the antibacterial activity of terpenoids depends on the type of alkyl substituent incorporated in a non-phenolic cycle. The presence of a carbonyl function in the terpenoids’ chemical structure also increases the antibacterial properties. Another aspect which influences the bioactivity of essential oils is their stereochemistry and the introduction of a double bond which increases the activity against Gram-negative bacteria [9].

The mechanisms of antimicrobial action identified in the EOs are the inhibition of microorganisms’ growth (food safety) and the control of natural spoilage processes (food preservation) [10].

EOs are complex mixtures of bioactive compounds, phytochemicals such as monoterpenes, sesquiterpenes, terpenoids, alcohols, aldehydes, ketones, phenolics, esters, and other complex aromatic and aliphatic compounds. These phytochemicals possess antibacterial, antifungal, anti-parasitic, and antiviral properties [11,12,13]. The different biological effects of EOs are due to the concentration and proportion of their chemical constituents. For example, EOs with higher concentrations of terpenoids exhibit higher antifungal activity compared with EOs rich in monoterpenes and sesquiterpenes [11]. EOs rich in thymol and carvacrol show higher membrane-damaging activities in bacteria than those less rich in phenolics. 

In view of the published data on the antimicrobial efficacy of EOs, the following general ranking (in order of decreasing antibacterial activity) can be made: oregano > clove > coriander > cinnamon > thyme > mint > rosemary > mustard > cilantro/sage.

The antimicrobial properties of EOs are mediated via several mechanisms: (a) EOs disrupt the permeability barrier in cells and induce various morphological and physiological changes (lipophilic moieties of the EOs attack the cytoplasmic membrane, leading to changes in membrane stability, hydrophobicity, fluidity, and fatty acid composition [14], and (b) EOs also disrupt proton pump function, destabilizing cell membrane architecture, which causes an uncontrolled flux of H^+^ ions, resulting in the inhibition of H^+^ ion-dependent movement of solutes across the membrane and the disruption of the intracellular pH.

Regarding concentrations, several studies have shown that the antimicrobial activity of essential oils in foods takes place at modest concentrations (200 µL/L), for example in *Thymus algeriensis* L. EO and *Rosmarinus officinalis* L. EO [15]. Espina et al. [16] also reported that the combination of low concentrations of individual constituents present in lemon essential oil (50–20 µL/L of carvacrol or citral) inactivated foodborne pathogens in fruit juices.

However, the incorporation of single EOs as antimicrobial agents in certain foods (for example in meat) may produce favourable conditions promoting the growth of undesirable microorganisms, due to possible changes in the ecology of the microorganisms [17]. Therefore, individual constituents of EOs may not be able to inactivate meat spoilage by microorganisms. In this situation, a combination of different EOs may help by causing an additive antimicrobial effect. For example, Gutierrez et al. [18] demonstrated that a combination of *Origanum majorana* and *Thymus vulgaris* EOs had an additive effect on meat spoilage.

Essential oils exhibit two main characteristics: low risk of resistance by pathogenic microorganisms and low toxicity for humans [19]. 

The development of natural products as preservatives of meat currently focusses largely on essential oils. Regarding antimicrobial activity, the use of essential oils in meat products is a strategy for the reduction of synthetic antioxidants. However, the effects of *R_EO_* depend on the characteristics of the food matrices and on its concentration [20].

Several benefits of using this EO as a food preservative have been identified: extended shelf life, hypoallergenicity, the improvement of aroma and taste, and possible health benefits to consumers due to its antioxidant and anticancer effects [21]. Although there is considerable literature exploring the antioxidant and antimicrobial properties of EOs derived from numerous plants, for most EOs, the in vitro results do not easily translate into actual use as meat preservatives. EOs are more effective when they come into direct contact with organisms; however, direct contact affects the organoleptic properties of the meat. Essentials oils tend to have an intense aroma even at low concentrations.

Another negative aspect is that different mould and yeast (*Fusarium*, *Mucor*, *Candida* and *Torulopsis*) or bacteria species (*Pseudomonas*, *Acinetobacter-Moraxella*, *Enterobacteriaceae*) cause spoilage of meat. Since EOs possess antibacterial, antifungal, anti-parasitic, and antiviral properties, their use may be part of a good strategy to control meat spoilage. Despite this, they may also represent some disadvantages: EOs may eliminate certain types of bacterial populations and produce favourable environments for the promotion of virulence and the growth of undesirable microorganisms. Additionally, strong aromas and flavours associated with EOs may cause negative organoleptic changes which will affect the consumer acceptability of the meat. Finally, it also must be noted that some EO phytochemicals interact with meat constituents and, in this way, reduce the efficacy of the EOs’ meat spoilage prevention.

### 2.2. Antifungal Activity

Fungal infection can be very dangerous for humans, producing mycotoxins and reducing or destroying the nutritive value of foodstuffs, grains, and leaves during storage. Numerous EOs and their constituents have been investigated for their antimicrobial properties against different fungi [4]. Antifungal activity is estimated in terms of growth inhibition, and the order of activity is phenols > cinnamic aldehydes > alcohols > aldehydes > ketones > ethers > hydrocarbons [20].

Specifically, the antifungal activity of phenolic components increases with the steric hindrance of the molecule (p-n-propylphenol < thymol < isoeugenol < eugenol) [22]. Pelczar et al. [23] reported that the addition of alkyl groups to the benzene ring of phenol increases the antifungal property, because a certain degree of hydrophobicity of aromatic aldehydes and phenolic components seems necessary to show an optimal antifungal effect.

### 2.3. Anti-Angiogenic and Anti-Tumoural Potential of Essential Oils

Angiogenesis is a normal and vital physiological process which, in a non-pathological situation, involves the growth of new blood vessels from pre-existing ones. However, this process also participates in the fundamental step of tumour transition from a dormant to a malignant state. Therefore, angiogenesis inhibitors are used in the treatment of cancer.

Recently, certain EOs have been proposed as non-toxic anti-angiogenic agents. Bonstancioglu et al. [24] reported the capacity of *Origanum* EOs to inhibit cancer cell viability and angiogenesis [24]. The EOs’ constituents carvacrol [25] and thymol [26] have been reported to have cytotoxic effects on cancer cell lines. Liang and Lu [27] reported the pro-apoptotic activity of carvacrol, considered the main bioactive compound for pro-apoptotic and anti-angiogenic effects [25,26,27].

Many studies have indicated that intake of foods with high levels of flavonoids may have the potential to decrease the risk of certain cancers, such as pancreatic, breast, and colon cancers. The treatment of cancer cells is performed with drugs which induce cell cycle arrest or apoptosis. EOs act in chemoprevention and cancer suppression. EOs from different plants have been reported to have anticancer potential against mouth, breast, lung, prostate, liver, colon, and brain cancers, and even against leukaemia [25,26,27].

### 2.4. Antioxidant Activity

The use of EOs as natural antioxidants is a strategy of growing interest in the food industry, because consumers do not consider synthetic antioxidants healthy. The reason for introducing synthetic antioxidants into food is to prolong these products’ shelf life; therefore, as substitutes for these compounds, EOs’ addition in active packaging or direct mixing is an alternative by which to extend the shelf life of edible foods [28]. The EOs’ overall performance as antioxidants is, in fact, the result of the complex interplay among the EO components and the oxidizable material to be protected. An antagonistic and synergistic effect is also expected between individual components of EOs [29]. 

The capacity of EOs to act as antioxidants is due to the fact that phenols are chain-breaking antioxidants. They donate an H-atom from the phenolic hydroxyl group to peroxyl radicals (ROO which are responsible for the propagation of the oxidative radical chain). Therefore, they can slow down the peroxidation of unsaturated lipids.

When considering the antioxidant activity of different EOs, it is important to mention that the variety of the plants, the agronomic practices and the processing can affect the chemical profile and the effective inhibitory concentrations of EOs; Delaquis et al. [30] reported that antioxidant, antimicrobial, and sensory properties differed between EOs of different origins. A higher antioxidant capacity is shown when the EOs contain a fair number of components, such as γ-terpinene (cyclohexadiene), and fair amounts of phenolics [31]. EOs with a higher proportion of phenolic sesquiterpenes and/or monoterpenes have been recognized for their antioxidative capacity [32].

The antioxidant property of essential oils and their components has often been verified in vitro by physical–chemical determination. In this respect, Kulisic et al. [33] studied the oxidation of lard in the presence/absence of some EOs (*Origanum* and *Thymus*) or their components. These EOs showed a strong phenolic profile characterized by the presence of the phenolic monoterpenes thymol and carvacrol. The antioxidant capacity of the essential oils was lower in comparison to α-tocopherol, synthetic antioxidants (BHA and BHT), and ascorbic acid. Different cases of synergism between EO components were reported, for example oregano EO (containing 67% thymol + carvacrol and ~14% terpinene). In contrast, for *Thymus* EOs (80% thymol + carvacrol and ~5.5% γ-terpinene), an intermediate behaviour was reported. Regarding the application of essential oils in food systems, different studies have reported that EOs prevented unwanted oxidation and controlled oxidative spoilage in bakery products [34], fish [35], or lamb meat [36], pigs [37], bulls [38], and chicken [39,40].

## 3. Essential Oil of the Lamiaceae Family

### 3.1. Essential Oil Extracted from Rosemary (R_EO_)

The demand for essential oils from medicinal plants has increased in recent years, especially in the case of oil from rosemary, which is used as a natural food preservative (listed by the European Food Safety Authority and the US Food and Drug Administration). 

The genus *Rosmarinus* includes popular herbs of the Lamiaceae family with potent antioxidant activity. It has aromatic and medicinal species (for example *Rosmarinus officinalis* L.) with antimicrobial and antioxidant activities [41,42,43].

Rosemary is a plant which can be used in different ways as an aromatic medicinal and as a culinary plant. Accordingly, it is grown all over the world, particularly in Spain, France, England, Russia and Portugal. This genus is extremely rich in essential oil, although there are large intraspecies differences in terms of the yield and quality of the essential oil produced.

Rosemary is the only herb commercially available for use as an antioxidant in Europe and the United States [21]. Its extract contains antioxidant compounds, the most active being phenolic diterpenes, such as carnosic acid, carnosol, rosmanol, epirosmanol, isorosmanol, methylcarnosate, and rosmarinic acid. The biological activities of *Rosmarinus officinalis* essential oil (*R_EO_*) have been reported in numerous studies, mainly focussing on its antioxidant [44], antibacterial [45], and antifungal properties [46]. This plant has a high availability and low cost.

The antimicrobial effect of *R_EO_* has been reported in several studies testing its antibacterial activity. Gomez-Estaca et al. [47] reported that rosemary essential oil totally inhibited the growth of *Salmonella choleraesuis*, *Shigella sonnei*, *Yersinia enterocolitica*, *Staphylococcus aureus*, *Bacillus cereus*, *Clostridium perfringens*, *Aeromonas hydrophila*, and *Photobacterium phosphoreum*. These bacteria are common food pathogens and bacteria contributing to food spoilage. In addition, Burt [34] also reported a good inhibitory in vitro effect of *R_EO_* against *Staphylococcus aureus*, *Bacillus cereus* and *Escherichia coli*. In general, Gram-negative bacteria are less sensitive to essential oils (due to the hydrophilic cell wall structures which block the penetration of hydrophobic components through the cell membrane) than are Gram-positive bacteria [48]. 

One example of incorporation of rosemary into meat was reported in 2013 by Sirocchi et al. [49]. These authors incorporated *Rosmarinus officinalis* EO at 4 (*w*/*w*) in active packaging of meat. This EO inhibited the increase of putrefaction products, such as cadaverine and the development of bacteria, such as *Enterobacteriaceae* and *Brochothrix thermosphacta*.

Along similar lines, Estévez and Cava [50] showed that the effect of *R_EO_* on the oxidative stability of frankfurters depended partly on the level of essential oil used and partly on the origin of the meat used to make frankfurters (made either from Iberian or non-Iberian pork). An antioxidant effect was observed when 150 ppm of *R_EO_* were added to the frankfurters made with non-Iberian pork, and no effect was observed on lipid oxidation with doses of 300 and 600 ppm. In contrast, rosemary essential oil inhibited the development of lipid and protein oxidation in a concentration-dependent manner in frankfurters made with Iberian pork.

The acceptance among consumers of the use of essential oils is growing, and more than 150 essential oils are listed as GRAS (generally recognized as safe) by the US Food and Drug Administration. However, the protective effects of natural antioxidants, such as essential oils, against protein oxidation have been little investigated, as recognized by Nieto et al. [39], who studied the mechanisms behind the protection of protein against oxidation by natural antioxidants in meat patties. For this study, two levels (0.05% and 0.4%) of rosemary essential oil were added to pork patties, and their effects on the protein oxidation occurring in the patties during storage were studied. The oxidative stability of the meat proteins was evaluated by the loss of thiols and the formation of myosin cross-links. These authors reported that essential oils of rosemary retarded protein oxidation. 

### 3.2. Essential Oil Extracted from Oregano (O_EO_)

*Origanum vulgare* is one of the most economically important species of the genus *Origanum*, found mainly on the Mediterranean coast.

The main compounds identified in the different essential oil of *Origanum* (*O_EO_*) are carvacrol and thymol, which are responsible for the characteristic odour and the antimicrobial and antioxidant activity. These compounds can donate hydrogen atoms to free radicals and convert them to more stable non-radical products. The oil’s effects are the result of various possible mechanisms of action involving transition-metal chelating activity, singlet-oxygen-quenching capacity, and free-radical scavenging activity [51].

Carvacrol and thymol provoke alterations in the fungi hyphal morphology and aggregates, resulting in lyses [52]. Different components of essential oil (such as terpenoids) could also interfere with the phospholipid bilayer membranes of bacteria, causing antimicrobial effects [53].

The properties of the *O_EO_* mentioned above make them natural additives for potential use in the food industry.

The addition of *O_EO_* into the animal diet or their addition as ingredients in meat products are both excellent strategies to improve the dietary value of and obtain a better oxidative stability and longer shelf life for food. In fact, the addition of *O_EO_* to animal food was reported to provide better lipid stability and to extend the shelf life of turkey, eggs, beef, and fat [54,55].

Nieto et al. [28] studied the effect of two concentrations (0.05% and 0.4%) of *O_EO_* on protein oxidation in pork patties during storage. The results of this study reported that these amounts of *O_EOs_* were able to protect the patties against thiol loss in chilled raw pork throughout the nine-day storage. 

### 3.3. Essential Oil Extracted from Thyme (T_EO_)

Thymus (thyme) belongs to the Lamiaceae family, which comprises about 110 species. It has been used for its medicinal effects (digestive, anti-inflammatory, etc.) and is one of the most valuable food preservatives in the food industry [56]. 

*T_EO_* contains more than 60 components, most of which possess important beneficial effects; for example, antiseptic, carminative, antioxidant, and antimicrobial properties. The most important compounds of *T_EO_* are the phenols thymol (68.1%) and carvacrol (3.5%), which constitute the major and most active constituents, as well as the monoterpene hydrocarbons *p*-cymene (11.2%) and γ-terpinene (4.8%), which are known to have antioxidant properties and antimicrobial activity. The antibacterial properties of these compounds are, in part, associated with their lipophilic character, leading to their accumulation in membranes and to subsequent membrane-associated events, such as energy depletion [57]. (Poly)phenolic compounds are characterized by having redox properties, which allow them to act as reducing agents, hydrogen donors, singlet oxygen quenchers, and metal chelators. Several researchers have carried out assessments of antioxidant activity based on the DPPH assay in different *Thymus* species. These studies showed that the chemical structure of the phenolic compounds of *T_EOs_* allows them to donate hydrogen to free radicals and explains their antioxidant activity [58]. In addition, when human intestinal Caco-2 cells were exposed to micelles resulting from beef patties treated with 10% herbal *Thymus* extracts and partial digestion, the cellular reduced glutathione content significantly increased and prevented H_2_O_2_-induced depletion in those cells [59].

## 4. Conclusions

Essential oils have great potential as food preservatives, due to their antioxidant and antimicrobial properties. Taking into account these properties, some essential oils may be considered for applications to different food systems such as meat, dairy, oils, and dressing. Many previous studies focussed on technological rather than nutritional aspects, and most studies on the biological activities of EOs were carried out in vitro, but not in vivo. Therefore, more studies in human systems are necessary to understand the effects of EOs on human health and their potential contribution to the nutrient and safety characteristics of food systems. It is critical to understand the effect of EOs and to optimize their combinations for use in food preservation to better exploit their possible synergistic effects in preventing both lipid oxidation and spoilage by pathogenic organisms. These considerations warrant the search for new medicinal plant species with high phenolic compound contents for beneficial applications in the food industry.

## Abbreviations

REO	Rosemary essential oil
OEO	Oregano essential oil
TEO	Thyme essential oil
